# Current Understandings on Magnesium Deficiency and Future Outlooks for Sustainable Agriculture

**DOI:** 10.3390/ijms22041819

**Published:** 2021-02-12

**Authors:** Ahmad Hassan Chaudhry, Shafa Nayab, Syed Bilal Hussain, Muqarrab Ali, Zhiyong Pan

**Affiliations:** 1 College of Horticulture and Forestry Sciences, Huazhong Agricultural University, Wuhan 430070, China; ahmadhassanch@webmail.hzau.edu.cn; 2 Department of Horticulture, Muhammad Nawaz Shareef University of Agriculture, Multan 60000, Pakistan; shafa.nayab123@gmail.com (S.N.); bilal.hussain@mnsuam.edu.pk (S.B.H.); 3 Department of Agronomy, Muhammad Nawaz Shareef University of Agriculture, Multan 60000, Pakistan; muqarrab.ali@mnsuam.edu.pk

**Keywords:** Mg deficiency, plants, cellular signaling, Mg transporters, Mg homeostasis

## Abstract

The productivity of agricultural produce is fairly dependent on the availability of nutrients and efficient use. Magnesium (Mg^2+^) is an essential macronutrient of living cells and is the second most prevalent free divalent cation in plants. Mg^2+^ plays a role in several physiological processes that support plant growth and development. However, it has been largely forgotten in fertilization management strategies to increase crop production, which leads to severe reductions in plant growth and yield. In this review, we discuss how the Mg^2+^ shortage induces several responses in plants at different levels: morphological, physiological, biochemical and molecular. Additionally, the Mg^2+^ uptake and transport mechanisms in different cellular organelles and the role of Mg^2+^ transporters in regulating Mg^2+^ homeostasis are also discussed. Overall, in this review, we critically summarize the available information about the responses of Mg deficiency on plant growth and development, which would facilitate plant scientists to create Mg^2+^-deficiency-resilient crops through agronomic and genetic biofortification.

## 1. Introduction

**Figure 1:**
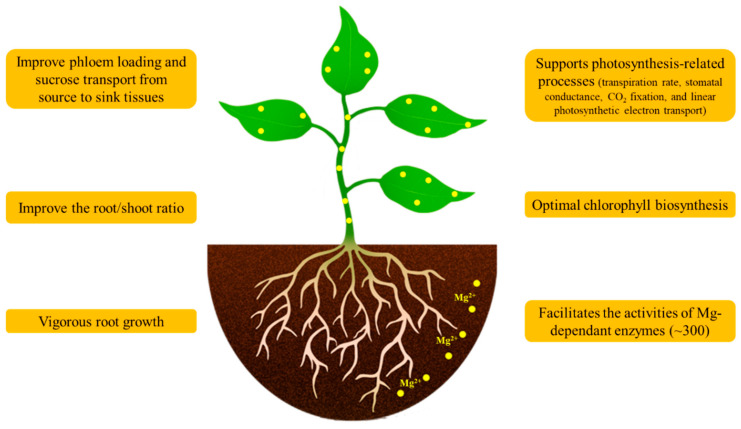
Physiological and morphological growth-dependent Mg functions in plants.

Magnesium (Mg) is one of the nine essential macronutrients that is used in large quantity by plants for their growth and reproduction [1,2]. In plant cells, Mg performs several physiological functions (Figure 1). Mg serves as the key atom of chlorophyll, where it acts in pigment-protein complexes to gather photons in photosystem I (PSI) and photosystem II (PSII) [3,4,5]. Apart from light absorption in the chlorophyll tetrapyrrole ring, Mg is also involved in CO_2_ assimilation reactions in the chloroplast [6]. Ribosomes are the macromolecular structures that are responsible for protein biosynthesis [7]. A large proportion of the Mg (about 75%) in leaf cells is associated either directly or indirectly with protein biosynthesis via its role in the ribosomal structure and function [8]. The dexterous type of ribosomes requires the blend of two subunits, compelling Mg to frame a bridge between these subunits; otherwise, these ribosomal subunits are unstable at low Mg^2+^ concentration (<10 mM) [9].

Mg has several other functions; particularly, it acts as a cofactor and allosteric modulator for more than 300 enzymes, including carboxylases, phosphatases, protein kinases, RNA polymerases, and ATPases [10]. Moreover, Mg is also involved in photophosphorylation, photosynthetic CO_2_ fixation and metabolism, and photoassimilates partitioning and utilization [2,11]. As it has well-known roles, Mg is important to plants. The “metabolic Mg pool” is mainly positioned in the cytoplasm and chloroplast; it is subject to strict regulatory processes and is specifically adapted to the actual metabolic needs while the vacuole acts as the storage compartment. The demand for “metabolic” Mg can thereby be satisfied and sustained through the import or export of Mg from the vacuole [12].

Mg deficiency is a common nutritional disorder in plants and a widespread problem affecting crop productivity and quality [13]. About 90%–98% of the soil Mg is combined in the crystal lattice structure of minerals and not directly available to plants [14]. The only existing form of Mg for uptake is Mg^2+^, which has the lowest ionic radius and the biggest hydrated radius among different cations [15]. This unique chemical property creates a weak bond between Mg^2+^ and negatively charged soil colloids as well as root cell borders, which favor the deficiency of interchangeable Mg from the soil. Over the last two decades, several studies were conducted to investigate the impact of Mg deficiency on plant growth and development, whereas few recent reports have focused on the Mg transport mechanism in plant cells, and it is still largely unknown. More effort should be paid to the shortage of Mg in soil and its impact on plants. In this review, we aim to recapitulate current knowledge about the causes of Mg deficiency, physiological and biochemical changes, and the response of Mg transporters, Mg uptake and distribution, and the possible solutions for improving Mg^2+^ utilization in plants to provide a better understanding of the Mg homeostasis in plants and, ultimately, to improve plant growth and development.

## 2. Causes of Mg Deficiency and Symptoms in Plants

Mg deficiency is a common nutritional disorder in plants that inhibits plant growth and development and eventually affects the yield and quality [2,16,17]. Mg scarcity is becoming an important concern in an intensive cropping system in which the soils are only fertilized with N, P, and K. It is also a critical issue in highly weathered soils, where it is subjected to potential leaching and interaction with aluminum (Al) [2].

Since Mg has a large hydrated radius, it is weakly absorbed by highly weathered, acidic, and coarse soils. Therefore, these soils are found to be Mg deficit soils due to excess leaching of Mg nutrient, specifically in acidic soil with low cation exchange capacity [1,13]. In soils with limited fertility, leaching of Mg can be as high as 25 kg ha^−1^, while it can be increased up to 40–70 kg ha^−1^, depending upon numerous variables such as soil and crop type, environmental conditions, and drainage volume [1,18]. In calcareous soils, the existence of Ca and bicarbonates (HCO_3_^−^) in higher aggregates affects the uptake of Mg and results in Mg depletion [19], whereas in alkaline soils, Mg availability is reduced due to the formation of magnesium carbonate and gypsum [20].

The common symptoms of Mg deficiency are growth retardation and interveinal chlorosis on older leaves [2]. Normally, chlorosis begins in older leaves and then progresses to younger leaves [21,22]. Due to the fair mobility of elements, plants remobilize Mg from older leaves to younger leaves; that is the reason why the first visual deficiency symptoms usually appear on older leaves and then on newly expanded leaves. Normally, Mg concentration under 1–2 mg g^−1^ dry leaf weight is related to the initiation of chlorosis [23,24,25].

Several studies have identified constraints related to plant growth and development that vary among plant species due to the intensity of Mg deficiency. Initially, the effects of Mg deficiency comprise of impedance in sugar accumulation, which leads to starch accumulation and ignition of antioxidant mechanism before any noticeable effect on the activity of photosynthesis [21,24].

## 3. Changes Induced by Mg Deficiency in Plants

### 3.1. Plant Growth and Biomass Allocation

**Figure 2:**
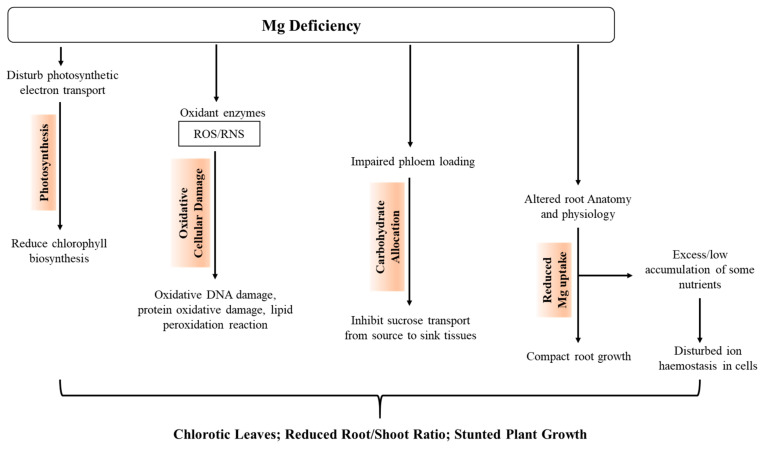
Physiological Impacts of Mg deficiency in plants.

Dwarf architecture and necrotic spots are the general symptoms of Mg deficiency in plants, which in result cause a decline of chlorophyll content and CO_2_ fixation, as well as impairment in carbon metabolism (Figure 2) [11,26]. Mg deficiency symptoms are well observed on aerial parts of plants, while the symptoms on root development and biomass distribution between root and shoot are variable [27]. Mg deficiency at germination or a young stage results in the severe reduction of root growth. In such circumstances, reduction in dry matter production was first detected in roots, then shoots, of clover [28], pepper [29], pine [30], and bean [31,32].

A transcriptome study in *Arabidopsis* indicated a far greater change in the number of genes in roots than that in leaves under Mg scarcity [27]. After 24 h of Mg supplementation, transcript patterns of one-fifth of the differentially expressed genes in leaves and half of genes in roots returned to normal levels. Moreover, Mg-deficiency responsive genes in leaves changed gradually after 8 h and changed even more after one week [27]. The results obtained from this study create a better understanding of changes in Mg availability that is translated into adaptive responses in the plants.

### 3.2. Photosynthetic Activity

Mg depletion was found to affect the activity of rubisco, which results in the reduction of the photosynthesis rate (Figure 2) [25,30,33]. In several plant species, decreases in photosynthetic capacity and net CO_2_ assimilation have been noticed [17,24,25,33,34,35,36,37]. Moreover, the structure and the partial photochemical activities of PSI, PSII, and F_v_/F_m_ ratio (greatest quantum efficiency of PSII) were also found to be affected by Mg deficiency [17,35,38]. These reports concluded that the decline of PSII activity is due to a deprivation of PSII antenna or an alteration in photosystem stoichiometry in favor of PSI, which results in an enhanced chlorophyll a/b ratio. Henceforth, an increased chlorophyll a/b proportion is quite often seen under Mg deficit conditions. However, the F_v_/F_m_ and other fluorescence parameters were not influenced in Mg-deficient *Helianthus annus* plants [39].

### 3.3. Chlorophyll Degradation

Mg occupies the key position in the chlorophyll, expectedly the effect of Mg insufficiency on overall chlorophyll concentration in several plant species, as reported in [11,17,24,40,41,42]. Chlorophyll catabolism can be considered as a strategy of Mg-deficient plants to dechelate Mg from pigment molecules [43]. In *Arabidopsis* and other plant species, *NYE1* was found to encode a chloroplast-confined protein that can initiate chlorophyll deterioration through senescence [44,45], and *MRP3* encodes an ABC carrier that has chlorophyll catabolites and glutathione conjugates transfer activity [46]. Later, global transcriptome studies showed that *NYE1* and *MRP3* were upregulated upon Mg scarcity [27].

### 3.4. Photosynthates Partitioning from Source to Sink

Accumulation of sugars and amino acids in the source organs usually inhibit their growth by causing a reduction in photosynthetic rate and chlorotic appearance (Figure 2). Photosynthate accumulation in source leaves maybe because of (i) structural impairment and destabilization in the phloem tissues [47], (ii) a decline in sink strength [7], or (iii) impaired phloem loading [11,32,48].

The inhibition of sucrose supply from Mg deficient leaves is primarily caused by the decline in Mg-ATP concentration at the phloem loading sites [21]. Mg-ATP is critical for the proper operation of H^+^-ATPase, and the growing evidence indicates that Mg-ATP is the chief complex of ATP in the cells [49]. H^+^/sucrose co-transporter is responsible for the catalyzation of sucrose loading into the phloem, whose activity necessitates a proton gradient maintained by an H^+^-ATPase situated in the plasma membrane of the sieve tube cells [21]. Under long-term deficient conditions, lower leaves also accumulate higher starch contents, which results in the restricted growth of root and taproot. Hence, carbon accumulation in source leaves is the major change induced by Mg deficiency in plants.

### 3.5. Ultrastructure Alteration and Oxidative Damage

The appearance of the chlorosis and necrosis symptoms in plants referred to the damage in chloroplast ultrastructure. A connection between carbohydrate accumulation and necrosis appearance was often endorsed because of a gathering of bigger starch grains with disintegrated thylakoids [22,50]. Under Mg shortage, chlorosis starts to progress due to the reduction in electron transport, which results in the impairment of CO_2_ fixation and induction of ROS generation (Figure 2) [51]. Damage of cell elements, such as membrane lipids, proteins, and nucleic acids, can be caused by these ROS, resulting in metabolism disruption [52]. Meanwhile, the antioxidative defense system is activated to scavenge ROS, which protects cells from oxidative damages [17,29,53]. For example, accumulation of malondialdehyde was reported in citrus [17,54], coffee [55], *Mentha pulegium* [56], maize [57], rice [58], and *Sulla carnosa* [34] under Mg depletion conditions. Moreover, an induction in the activity of SOD has been reported in mulberry [53], rice [58], and bean [59]. Under Mg scarcity conditions, the activities of other enzymes such as CAT and POD may also be increased. In some cases, it was noticed that the activity of CAT was decreased or remained constant [53,57], which indicates that the antioxidant system in plants can be destroyed by severe deficiency.

## 4. Mg Uptake and Transport in Plants

The attainment of Mg in plants from soil is generally governed by two fundamental processes: the abundant flow, which involves the passive movement of Mg^2+^, and diffusion, which involves the movement of Mg^2+^ from higher concentration to lower concentration. Plants have developed high-efficiency transport frameworks for Mg uptake, storage capacity, and movement to maintain a higher concentration (typically mM) in a given tissue. Mg is mostly transported into the cell for various biological processes. The accumulation of Mg^2+^ is higher in the chloroplast (0.5–2 mM [60]) and the mitochondria (2–4 mM [61]), followed by the cytosol (0.2–0.4 mM [49]). Therefore, Mg transporters are required to assist the accumulation of Mg in the corresponding parts of the cell. In addition, the vacuole is an enormous Mg pool to keep the cytosolic Mg equilibrium. Additionally, it needs carriers to encourage the Mg trade with cytosol.

### 4.1. Identification, Characterization, and Physiological Significance of Mg^2+^ Transporters Gene Families

Specific transporters are believed to function in Mg^2+^ transport across the membrane, which maintains homeostasis in the plant cell. CorA is the dominant transport system that was first identified in bacteria [62]. The movement of Mg^2+^ in and out of the cell is controlled by the members of the CorA family by utilizing a range of divalent cations as substrates [63]. Five CorA family genes arbitrate uptake across the plasma membrane, uptake into the mitochondria, and efflux from vacuoles. Namely, aluminum Resistance 1 (ALR1) and aluminum resistance 2 (ALR2) function for the Mg uptake across the plasma membrane into the cell [64,65]. A mitochondrial Mg transporter, named MRS2, was identified due to its capability of correcting a defect in RNA splicing, demonstrating the important role of Mg in cellular biochemistry [66,67]. Another mitochondrial Mg transporter, named LPE10, was identified based on the sequence similarity with CorA [66]. The fifth CorA homolog, named MNR2, has been characterized to acquire Mg that is stored in the vacuole [68].

Largely known plant Mg^2+^ carriers MGT (Mg transporter)/MRS2 genes [65,69] belong to the superfamily of CorA-type membrane transporters [70]. The first *Arabidopsis* CorA homologs were identified in expressed sequence tag by homology to yeast MRS2. *Arabidopsis* MRS2-1 (AtMRS2-1) functionally complemented the yeast MRS2 mutant [71]. The MGT family contains the diversity in functions throughout the plant life cycles, such as in root growth, pollen development, leaf photosynthesis, and defensive response to abiotic stress [72,73,74,75,76,77]. In *Arabidopsis*, ten CorA superfamily *AtMGT* genes were found; among them, *AtMGT8* is a pseudogene, and some are classified into high affinity (*AtMGT1* and *AtMGT10*), low-affinity (*AtMGT3*, *AtMGT7*, and *AtMGT9*), and dual-affinity (*AtMGT5*) individuals by their affinities for Mg^2+^ [65,71]. Li and Tutone [65] stated that MGT family members may likewise transfer divalent cations other than Mg^2+^, such as Ni^2+^, Co^2+^, Fe^2+^, Mn^2+^, and Cu^2+^. However, a conserved Gly-Met-Asn (GMN) tripeptide motif at the end of the first transmembrane domain has a higher affinity for Mg^2+^ than that of other cations, and the mutation for this motif abates the transportation of Mg^2+^ [78].

Additional gene families, other than CorA homologs, are also involved in Mg transport such as MgtA, MgtB, and MgtE [79,80]. Typically, MgtA and MgtB are P-type ATPases that have 10 transmembrane segments [81,82,83,84], whereas MgtE is a distinct Mg transporter that has five transmembrane helices [85]. MgtA and MgtB are highly induced by low Mg conditions through the Mg^2+^ regulated PhoP/PhoQ two-component systems [86]. Unlike the CorA, MgtA and MgtB actively mediate Mg influx. Moreover, the expression of the MgtE-encoded gene is controlled by an Mg^2+^ sensing riboswitch [87]. MgtE can transport Mg^2+^ and Co^2+^ [85] whereas MgtA and MgtB transport only Mg^2+^ and Ni^2+^ [83].

In recent years, the participation of Mg transporters has been identified in the development of pollen and male fertility. AtMGT5, the mitochondrial membrane-localized protein and two ER target proteins, AtMGT9 and AtMGT4 are vital for normal production of pollen. In *Arabidopsis*, the disruption of *AtMGT4*, *AtMGT5*, or *AtMGT9* considerably downshifted the pollen development, whereas high expressions of both *AtMGT5* and *AtMGT9* resulted in bicellular pollen [79,88,89]. Disruption of expression of these genes results in the insufficient supply for pollen mitosis and intine formation, which at last prompts the diminished number of mature pollen grains. AtMGT5 and AtMGT9 may act coordinately to transfer Mg from tapetum to the microspore, as both the AtMGT5 and AtMGT9 are plasma-membrane-localized Mg transporters, while *AtMGT4* is primarily expressed in pollen grains from the bicellular to the developed pollen grain, as it is confined to the endoplasmic reticulum. The involvement of AtMGT4 in pollen development is still unclear, but the pollen production by *AtMGT4*-RNAi transgenic lines is just about half sterile [77].

### 4.2. Mg Uptake, Distribution, and Homeostasis in Plants

To resolve the mystery of Mg behavior, Mg transport and distribution in some plant parts was visualized using the radioisotope ^28^Mg (with half-life 21 h). According to Kobayashi, Iwata [90], a better accumulation of ^28^Mg was observed in rice root soon after the absorption from the external solution. In *Arabidopsis*, within 15 h of root uptake, ^28^Mg was gradually transported to the upper part of the shoot with steady accumulation in the lower part of the inflorescence. This behavior suggests that hours after the root uptake, Mg^2+^ reaches the upper part of the inflorescence. With the discoveries of Mg transporters, knowledge about Mg uptake, distribution, and homeostasis has been advanced. AtMGT1 localizes to the plasma membrane; it is predominantly expressed in the root and vascular tissues, and in the trichomes of newly grown leaves [72]. Plasma-membrane-embedded subcellular localization of AtMGT6 appeared in the root cells, while the localization either at the chloroplast or the mitochondria was indicated in the shoot tissue. In rice, MGT family protein, OsMGT1, meditated Mg uptake by roots [91]; in addition, AtMGT6 in *Arabidopsis* was found to mediate Mg uptake by roots and tolerance to low Mg [92]. Nonetheless, knockout of *MGT1* in rice and silencing *MGT6* in *Arabidopsis* failed to prevent the accumulation of Mg in roots of both plants, indicating that, in addition to these carriers, other Mg carriers act in roots to encourage the Mg uptake [73].

#### 4.2.1. Mg Transporters Involved in Xylem Loading

Transfer of Mg from roots to shoots is performed through xylem loading. Moreover, the transporters that are responsible for the translocation of Mg from root to shoot have not been studied to a deeper extent. In *Arabidopsis*, Mg^2+^/H^+^ exchanger (MHX) was highly expressed in vascular tissues, which propose the role of MHX in xylem loading or recovery of Mg [93]. Two CorA-like homologs (*OsMGT2* and *OsMGT6*) in rice and *AtMGT9* in *Arabidopsis* [72] are likely to be involved in the xylem loading, mainly expressed in the vascular tissues of root elongation and maturation zones. After the translocation of Mg from root to shoot, it is delivered to different tissues of a plant with the preferential distribution to developing tissues [94].

#### 4.2.2. Chloroplast Localized Mg Transporters

As discussed earlier, Mg plays a key role in photosynthesis; thus, Mg is preferentially transported into the chloroplast. MGT10, also named MRS2-11, was identified during the complementation screen of the yeast ALR1/ALR2 mutant [65]. AtMGT10 is localized to the chloroplast envelope membrane and is enriched in leaf [95]. The expression pattern of *AtMGT10* showed a circadian rhythm, which suggests its involvement in diurnal Mg homeostasis in chloroplast stroma where the photosynthetic enzyme action is directed by *AtMGT10*.

#### 4.2.3. Tonoplast Localized Mg Transporters

Part of the cellular Mg^2+^ is bound to the cell wall or sequestered in vacuoles, whereas free Mg^2+^ concentration in the cytosol might be lowered because Mg^2+^ is complexed with various molecules such as ATP. The free levels of Mg^2+^ in the cytosol must be strictly regulated due to Mg^2+^ effect on photosynthesis and membrane ionic currents. Thus, the ionic balance is required to continue the cellular processes.

Vacuole is the main organelle that determines Mg^2+^ homeostasis in the cytosol and the chloroplast [96]. It has been reviewed by Shaul [97] that vacuolar Mg^2+^ is important for the cation-anion balance and turgor regulation of cells. MHX (Mg^2+^/H^+^) is the first cloned Mg^2+^ transporter in plants, AtMHX, encoded by a single gene in *Arabidopsis*. MHX is confined in the vacuolar membrane of xylem parenchyma cells [93] that improve xylem loading in the roots and Mg^2+^ distribution at sink organs [97]. Surprisingly, tobacco (*Nicotiana tabacum* L.) plants indicated necrotic injuries and apical burnings upon development under raised Mg^2+^ or Zn^2+^ conditions, and the expression of *AtMHX* was accordingly inhibited by high Mg^2+^, although no change was observed in the mineral content between transgenic and normal plants treated with elevated levels of Mg^2+^ or Zn^2+^ [98,99]. These results indicated that the necrotic lesions in transgenic tobacco are not due to high Mg^2+^ and Zn^2+^, but because of an imbalance of proton in the cell.

In *Arabidopsis*, MGT2 and MGT3 are mesophyll-abundant and tonoplast-confined transporters, which aid in more Mg accumulation in the vacuole. Knockout of either *MGT2* or *MGT3* lowered the mesophyll-specific Mg accumulation, while the triple knockout lines of MGT1/2/3 showed severe developmental downshift under Mg-deficient conditions [100].

### 4.3. Factors Influencing Mg Homeostasis

The availability of Mg^2+^ to plants depends on various factors: distribution and chemical properties of source rock material, and its grade of weathering and site-specific climatic and anthropogenic factors. Mg^2+^ is exceptionally sensitive to discharging, which is considered as a crucial factor affecting Mg^2+^ availability for roots. Mg^2+^ leaching occurs usually in acidic soils (pH < 4.5, high H^+^/proton) with reduced cation-exchange capacity, and about 70% of the arable land on Earth is acidic. MgCO_3_ development and excess accumulation of Ca^2+^, K^+^, and Na^+^ in soluble soils likewise decreases Mg^2+^ accessibility to crops. Drought affected soil and some Al toxic soils likewise hinder Mg^2+^ retention by roots. Among a few contending elements in soils, such as K, Ca, Al, and NH_4_, Mg^2+^ is the least taken-up nutrient. Raised temperature and high precipitation in tropical areas also lead to Mg^2+^ leaching and lessens the balance between plant Mg^2+^ concentration and Mg^2+^ availability. Moreover, acidic soils impede Mg^2+^ uptake in plants by allowing higher leaching rates of Mg^2+^ and higher concentrations of Al and Mn.

## 5. Mg Stresses Signaling in Plants

There has been little research on signal transduction responding to the Mg deficiency in plants. In general, the signaling process may be related to respiration block [101], leaves to root transport of sugar via the phloem; this improves starch accumulation in leaves and resulting in the inhibition of photosynthesis, increase the cellular ROS level, and consequently restraining the plant growth [24]. However, those findings are too ambiguous to disclose the signaling in plants response to Mg deficiency.

In plants treated with Mg deficiency, no change in ABA content was observed, although half of the ABA-responsive genes were upregulated in the leaves [26], suggesting that the factors responding to ABA are involved in the ABA-independent signaling process [102], and according to Hermans and Vuylsteke [27], those ABA-responsive genes oscillate following the circadian clock. ABA and auxin are also involved in the regulation of AtMHX. This might be due to the following two reasons: first, the presence of an ABA response element (ACGTGTC) and the auxin response element (ACTTTA) in the promoter region, and second, the existence of a repetitive genomic element of 530 bp that functions as an enhancer in its leader intron [103,104,105]. Ethylene also plays a key role in Mg deficiency, as plants treated with Mg deficiency produced twice as much ethylene as compared to those treated with Mg sufficiency treatment, and the mRNA levels of the 1-aminocyclopropane-1-carboxylic-acid-synthase (ACS)-encoding genes (*ASC2*, *ASC7*, *ASC8*, and *ASC11*) in the biosynthetic pathway were enhanced accordingly [27]. Similar to ABA-responsive genes, ethylene production also follows a circadian rhythm, and the expression of *ACS8* is regulated by *CCA1* (circadian clock associated 1) and *TOC1* (timing of cab 1) [16]. Here, the phase of *CCA1* and late elongated hypocotyl (*LHY*) expression was delayed in Mg deficient plants grown in light/dark cycles. The effects of Mg deficiency on circadian clock genes expression might be due to impaired sugar production or partitioning, and sugar plays some roles in circadian entrainment [106]. Moreover, it might be due to other energy-dependent processes.

CKX (cytokinin-degrading cytokinin oxidase/dehydrogenase) gene is a negative regulator of root growth. Upon Mg-deficient conditions for both long and short time, transgenic plants (W6:*CKX1*) retained 15%–60% more chlorophyll as compared to the corresponding wild types leaves. However, the MGT genes exhibited a significantly decreased expression level in transgenic roots as compared with control, suggesting that the increased content of Mg^2+^ in transgenic leaves and their higher chlorophyll content may not have caused by the known Mg^2+^ transporters [102,107]. Reactive oxygen species (ROS) and cytosolic Ca^2+^ were confirmed as signaling factors responding to Mg deficiency [108], in agreement with a previous hypothesis that antioxidation is an early response to Mg deficiency induced by ROS [21,27].

## 6. Effects of Mg on Other Nutrients Uptake Behavior

Mg^2+^ and other ions have two kinds of interactions with each other. If two or more nutrients participate together to create an overall improved and well-developed physiological state, it is known as synergism, while the excess of one nutrient inhibiting or reducing the uptake of other nutrients is known as antagonism. Both synergistic and antagonistic interactions depend on soil type, soil physical properties, soil pH, ambient temperature, and the proportion of participating nutrients. In addition, there is an involvement of a highly controlled selective process in the uptake of nutrients by plants, which is the reason behind the different ratios of nutrients inside the plants [109].

The excessive application of K and ammonium (NH_4_^+^), especially in sandy soils, often increases the chances of Mg scarcity [20]. Under Mg-deficient conditions, other nutrients (Ca, K) have strong antagonistic uptake behavior, which results in relative or absolute excess of Ca^2+^, H^+^, NH_4_^+^, and Al^3+^ [110]. Mg concentration and uptake was reduced in roots and leaves of rice plants when K concentration was increased in plants, but under the low supply of K, there was no effect on Mg concentration and uptake, which recommends that the opposing impact of K on Mg uptake was clearer than that of Mg on K uptake [23]. Similar effects were also observed by Farhat and Rabhi [111] that Mg and Ca concentrations were significantly decreased in all parts of safflower when plants were supplied with a higher amount of K (60 mM KCl), while under Mg scarcity, no effect on K concentration was noticed. Gransee and Führs [1] revealed that the blockage of unspecific Mg transporters can be brought about by the high accessibility of K in soil or rhizosphere. Thereby, two procedures were proposed for the uptake of K: the high-affinity transport system and the low-affinity transport system [112].

The availability of Mg can modify the uptake of Ca and K, while Ca and K can restrict the translocation of Mg from roots to shoots under insufficient Mg supply [113]. According to Lasa and Frechilla [39], when sunflower plants were grown under the Mg-deficient conditions, greater uptake of Ca and K was observed. A similar phenomenon was also found by Hermans and Johnson [24]; they noticed a marked boost of Ca in roots and petioles and K in all parts of sugar beet plants. In onion [114] and citrus [115,116,117], the antagonistic effects of Mg on Ca and K have also been reported. Moreover, Ye and Chen [117] also observed the most striking effects in the form of a large increase in Mn and Zn concentrations in the leaf blades of *Citrus sinensis*, proposed the existence of antagonistic interaction between Mg, Mn, and Zn. However, in *Medicago sativa*, the antagonistic action of Mg on Mn uptake hardly existed [118], which suggests that the antagonistic interaction and competitive effects of Mg on Mn vary depending on plant species, the nutrient concentration of the medium, and the cultivation type.

The synergetic effects of Mg^2+^ on K^+^ ions were noticed in rice plants [23]. It was further found that biomass yield and the photosynthetic rate were improved in plants due to Mg supply and low feed of K. An improvement of K uptake and its movement from roots to shoots was associated with the Mg supply. It is thought that Mg substitutes K in plant cells for a few of its roles that support the hypothesis proposed by Bedi and Sekhon [119], who suggested that absence of one cation can be compromised by another cation (Ca^2+^, K^+^, Mg^2+^).

## 7. Strategies to Enhance the Mg Use Efficiency in Plants

In agricultural production systems, Mg availability to crops depends upon the various factors such as soil texture, cation exchangeable capacity, site-specific climatic and anthropogenic factors, agronomic management practices, and the crop species itself [120]. Sufficient concentration of Mg in the soil is a key to ensure the vigorous crop growth and production, while the Mg deficient soils dramatically reduce the Mg absorption by crop roots, which is mainly because of low Mg content in source rocks, Mg losses by mobilization and leaching in the soil, Mg depletion due to the intensive crop production, and the cationic competition [121,122]. There is another important factor determining the crop productivity is soil acidity [123], closely associated with the nutrient deficiency such as K, Ca, Mn, P, and Zn, while the toxicity of Al and Mn [124,125,126] antagonizes the availability of Mg. In addition to this, the highly mobile nature of Mg^2+^ makes it vulnerable to discharge it from the root-zone due to the heavy rainfall [1], particularly in the acidic soils, resulting in reduced nutrient use efficiency and crop yield.

Two strategies are considered to increase the Mg^2+^ use efficiency in plants [127]. The first most used strategy is the agronomic biofortification, while the second one, today’s fast-accepted strategy, is the genetic biofortification that includes the breeding crops with higher Mg^2+^ contents and genetic modification of some genes related to Mg deficiency tolerance or Mg^2+^ uptake (Table 1).

Agronomic biofortification deals with sustaining plant growth and maintaining high Mg concentrations by applying Mg fertilizers, usually supplied as its sulfate, carbonate, or oxide slats. In addition, the use of magnesium ammonium phosphate (struvite) has recently received attention, as it has potential for sustainable P source for agriculture, and magnesium sulfate provides readily available Mg^2+^, where MgO behave as a slow-release fertilizer [156]. In some crops, the application of MgSO_4_ is common and Mg-deficient symptoms could be alleviated by foliar application of MgSO_4_ [157,158]. From a broader point of view, Mg fertilization improves the tomato yield (7.7–17.9 t ha^−1^) [159], grain yield in barley (8.6%) [160], fruit yield of hazelnut (51%) and total oil content (4.8%) [161]. In addition, the meta-analysis showed the increased yield of fruit, grass, tobacco, tuber, vegetable, cereal, oil crop, tea, and other crops with an average of 8.5%, when the reasonable amount of Mg (i.e., 94.1, 46.9, 54.1, 58.3, 43.5, 27.8, 47.2, 34.1, and 76.8 kg MgO ha^−1^, respectively) was applied [122]. Hence, the application of Mg^2+^ through fertilization and the foliar sprays may be useful to manage and correct the nutrient deficiency during the growing season.

Agronomic biofortification approaches can increase the content of bioavailable minerals to humans and livestock [158]. However, the agronomic efficiencies are dependent on the uptake or utilization of Mg across crop species [122]. Therefore, the future of sustainable farming demands optimized application methods to minimize environmental pollution and to save the economic margins. Thus, in the coming years, genetic biofortification may be a demanding and attractive strategy. Once an Mg^2+^ homeostasis target gene(s) is identified in some plants, it would be used for genetic biofortification by using forward genetics, reverse genetics, and screening of biparental crosses progeny in variety of crop species [127]. An early attempt was made by Deng and Luo [155] to modify the expressions of annotated Mg^2+^ transporters in plants. They were succeeded in increasing the Mg^2+^ content in the transgenic lines by 30% (Table 1). Furthermore, the utilization and absorption of Mg^2+^ can also be improved by modifying some factors in biosynthesis pathways of phytohormones, their responsive genes and QTL may also be valuable regarding discovering gene clusters responding to Mg stresses [127,162].

For maximum or optimal economic yield, farmers should ensure the adequate Mg supply, which at the same time will ensure optimal crop quality in virtually all cases. Recently, Guo [104] reviewed an article on Mg homeostasis and utilization and emphasized that manures and crop biofortification techniques need consideration of other ecological variables, including the adverse impacts due to dry season, warmth, and high radiation, low pH, and metal toxicity, and the genetic biofortification approaches might be extremely useful to feature the communication systems and find new avenues associated with the usage of Mg^2+^.

## 8. Conclusions and Future Prospects

With the development of the agriculture industry and the increase in the human population, Mg deficiency in plants is becoming a severe problem. Due to low sensitivity, the toxicity of Mg in plants is focused less than its deficiency. Additionally, plants have developed many physiological changes that make them less vulnerable to Mg toxicity, such as vacuolar compartmentation of Mg in plant cells [10], uptake of specific Ca at the root and transfer to shoot, and limitation of core Mg sequestration at the root level [163,164].

Over the last two decades, work has been performed to understand the response of Mg deficiency on plant growth and development. A recent review suggested that the discovery of Mg transporter might help in understanding Mg uptake and transport mechanisms [97]; however, it remains unclear. Moreover, Mg regulates the carbohydrate portioning via Mg-ATP complex. Apart from the role of Mg-ATP in phloem loading, Mg may play a direct or indirect vital role in the phloem loading [21], but it is not yet fully understood. Moreover, there is a need to identify the novel transcription factors, responsible for regulating Mg accumulation in plant cells.

## Figures and Tables

**Table 1:** Strategies to improve the Mg use efficiencies.

Agronomic Biofortification-Fertilization					
Crops	Fertilizer Used	Plant Parts Used	Improvement in Mg and Other Nutrient Concentration	Improvement in Yield/Biomass	References
Blueberry	MgSO_4_	Leaves, Fruits	✓	✓	[128]
Banana	MgSO_4_	Leaves	✓	Not examined	[129,130]
Citrus	NPK + MgSO_4_	Leaves, Fruits	✓	✓	[131]
Pumelo	MgSO_4_	Leaves, Fruit	✓	✓	[132]
Watermelon	Sulfate-Potassium-Magnesium	Fruit	✓	✓	[133]
Cabbage	MgSO_4_	Fruit	✓	Not examined	[134]
*Capsicum annum* L.	H_3_BO_3_ + MgSO_4_.7H_2_O	Leaves, Stem, Flower, Fruit	✓	✓	[135]
Carrot	MgO	Roots	✓	✓	[136]
Kale	MgSO_4_	Shoot	✓	✓	[137]
Potato	MgSO_4_, Calcined Magnesite, Kieserite	Tuber	✓	✓	[137,138,139,140,141]
Sugarbeet	MgSO_4_, Calcined Magnesite, Kieserite	Fruits/Roots	✓	✓	[137,141]
Onion	MgSO_4_.7H_2_O	Leaves, Bulbs	✓	✓	[114]
*Panicum maximum*	MgSO_4_.7H_2_O	Shoot	✓	✓	[142]
Pakchoi	Sulfate-Potassium-Magnesium	Leaves	✓	✓	[133]
Oolong Tea	MgSO_4_	Leaves	✓	✓	[143]
Wheat	MgSO_4_.7H_2_O	Leaves, Stem, Husk	✓	✓	[144]
Barley	MgSO_4_, Calcined Magnesite, Kieserite	Aerial parts of the plant	✓	✓	[137]
Maize	K_2_O + MgO	Shoot, Grain	✓	✓	[145]
Rice	MgSO_4_	Leaves, Grain	✓	✓	[146]
Sugarcane	MgSO_4_	Leaves, Stem	✓	✓	[147]
Sunflower	K_2_O + MgO	Leaves, Seeds/Grain, Stem	✓	✓	[148]
*Silybum marianum* (L.)	MgSO_4_, MgSO_4_ + Boron	Flowers	✓	✓	[149]
Lentil	MgSO_4_	Seeds	✓	✓	[150]
Peanut	MgSO_4_	Leaves, Fruit	✓	✓	[151]
Tobacco	MgSO_4_	Leaves	✓	✓	[152,153,154]
**Agronomic Biofortification—Mycorrhizal Fungai (*Glomus versiforme*)**					
Citrus	MgSO_4_	Leaves, Roots	✓	Not examined	[30]
**Agronomic Biofortification-Grafting**					
Watermelon	MgSO_4_	Leaves, Roots, Stem	✓	Not examined	[31]
**Genetic Biofortification**					
Tobacco	MgSO_4_	Leaves, Roots	✓	Not examined	[155]

## Data Availability

Not applicable.

## References

[1] Gransee A., Führs H. (2013). Magnesium mobility in soils as a challenge for soil and plant analysis, magnesium fertilization and root uptake under adverse growth conditions. Plant Soil.

[2] Cakmak I., Yazici A.M. (2010). Magnesium: A Forgotten element in crop. production. Better Crop..

[3] Jansson S. (1994). The light-harvesting chlorophyll ab-binding proteins. Biochim. Biophys. Acta Bioenerg..

[4] Mayland H., Greene L.W., Robinson D.L., Wilkinson S.R. Grass tetany: A review of Mg in the soil-plant-animal continuum. Proceedings of the 25th Annual Pacific Northwest Animal Nutrition Conference.

[5] Rissler H.M., Collakova E., DellaPenna D., Whelan J., Pogson B.F. (2002). Chlorophyll biosynthesis. Expression of a second chI gene of magnesium chelatase in Arabidopsis supports only limited chlorophyll synthesis. Plant Physiol..

[6] Hannaway D., Bush L.P., Leggett J.E. (1980). Plant Nutrition: Magnesium and Hypomagnesemia in Animals.

[7] Fischer E., Lohaus G., Heineke D., Heldt H.W. (1998). Magnesium deficiency results in accumulation of carbohydrates and amino acids in source and sink leaves of spinach. Physiol. Plant..

[8] Merbach W.H. (1988). Marschner, Mineral nutrition in higher plants, Academic Press, London, Orlando… (1986), 674 Seiten, 186 Abb., 257 Tabellen, Preis: $89,50; £49,95, ISBN: 0-12-473540-1. Zent. Mikrobiol..

[9] Noah J.W., Wollenzien P. (1998). Dependence of the 16S rRNA Decoding Region Structure on Mg^2+^, Subunit Association, and Temperature. Biochemistry.

[10] Hawkesford M., Horst W., Kichey T., Lambers H., Schjoerring J., Møller I.S., White P. (2012). Functions of macronutrients. Marscher’s Mineral Nutrition of Higher Plants.

[11] Hermans C., Bourgis F., Faucher M., Strasser R.J., Delrot S., Verbruggen N. (2005). Magnesium deficiency in sugar beets alters sugar partitioning and phloem loading in young mature leaves. Planta.

[12] Marschner H. (2012). Mineral Nutrition of Higher Plants.

[13] Aitken R., Dickson T., Hailes K.J., Moody P.W. (1999). Response of field-grown maize to applied magnesium in acidic soils in north-eastern Australia. Aust. J. Agric. Res..

[14] Senbayram M., Gransee A., Wahle V., Thiel H. (2016). Role of magnesium fertilisers in agriculture: Plant–soil continuum. Crop Pasture Sci..

[15] Maguire M.E., Cowan J.A. (2002). Magnesium Chemistry and Biochemistry. Biometals.

[16] Verbruggen N., Hermans C. (2013). Physiological and molecular responses to magnesium nutritional imbalance in plants. Plant Soil.

[17] Yang G.-H., Yang L.-T., Jiang H.-X., Li Y., Wang P., Chen L.-S. (2012). Physiological impacts of magnesium-deficiency in Citrus seedlings: Photosynthesis, antioxidant system and carbohydrates. Trees.

[18] Szabolc I. (1978). International Seminar on Soil Environment and fertility Management in Intensive Agriculture (SEFMIA). Agrokémia És Talajt..

[19] Farhat N., Sassi H., Zorrig W., Abdelly C., Barhoumi Z., Smaou A., Rabhi M. (2015). Is excessive Ca the main factor responsible for Mg deficiency in *Sulla carnosa* on calcareous soils?. J. Soils Sediments.

[20] Mayland H., Wilkinson S. (1989). Soil factors affecting magnesium availability in plant-animal systems: A review. J. Anim. Sci..

[21] Cakmak I., Kirkby E.A. (2008). Role of magnesium in carbon partitioning and alleviating photooxidative damage. Physiol. Plant..

[22] Farhat N., Rabhi M., Krol M., Barhoumi Z., Ivanov A.G., McCarthy A., Abdelly C., Smaoui A., Hüner N.P.A. (2014). Starch and sugar accumulation in *Sulla carnosa* leaves upon Mg^2+^ starvation. Acta Physiol. Plant..

[23] Ding Y., Luo W., Xu G. (2006). Characterisation of magnesium nutrition and interaction of magnesium and potassium in rice. Ann. Appl. Biol..

[24] Hermans C., Johnson G.N., Strasser R.J., Verbruggen N. (2004). Physiological characterisation of magnesium deficiency in sugar beet: Acclimation to low magnesium differentially affects photosystems I and II. Planta.

[25] Hermans C., Verbruggen N. (2005). Physiological characterization of Mg deficiency in *Arabidopsis thaliana*. J. Exp. Bot..

[26] Hermans C., Vuylsteke M., Coppens F., Craciun A., Inzé D., Verbruggen N. (2010). Early transcriptomic changes induced by magnesium deficiency in *Arabidopsis thaliana* reveal the alteration of circadian clock gene expression in roots and the triggering of abscisic acid-responsive genes. New Phytol..

[27] Hermans C., Vuylsteke M., Coppens F., Cristescu S.M., Harren F.J.M., Inzé D., Verbruggen N. (2010). Systems analysis of the responses to long-term magnesium deficiency and restoration in *Arabidopsis thaliana*. New Phytol..

[28] Bouma D., Dowling E., Wahjoedi H. (1979). Some effects of potassium and magnesium on the growth of subterranean clover (*Trifolium subterraneum*). Ann. Bot..

[29] Riga P., Anza M., Garbisu C. (2005). Suitability of the antioxidative system as marker of magnesium deficiency in *Capsicum annuum* L. plants under controlled conditions. Plant Growth Regul..

[30] Sun O.J., Payn T.W. (1999). Magnesium nutrition and photosynthesis in *Pinus radiata*: Clonal variation and influence of potassium. Tree Physiol..

[31] Cakmak I., Hengeler C., Marschner H. (1994). Partitioning of shoot and root dry matter and carbohydrates in bean plants suffering from phosphorus, potassium and magnesium deficiency. J. Exp. Bot..

[32] Cakmak I., Hengeler C., Marschner H. (1994). Changes in phloem export of sucrose in leaves in response to phosphorus, potassium and magnesium deficiency in bean plants. J. Exp. Bot..

[33] Ridolfi M., Garrec J.-P. (2000). Consequences of an excess Al and a deficiency in Ca and Mg for stomatal functioning and net carbon assimilation of beech leaves. Ann. Sci..

[34] Farhat N., Ivanov A.G., Krol M., Rabhi M., Smaoui A., Abdelly C., Hüner N.P.A. (2015). Preferential damaging effects of limited magnesium bioavailability on photosystem I in *Sulla carnosa* plants. Planta.

[35] Laing W., Greer D., Sun O., Beets P., Lowe A., Payn T. (2000). Physiological impacts of Mg deficiency in *Pinus radiata*: Growth and photosynthesis. New Phytol..

[36] Liang C., Xiao W., Hao H., Xiaoqing L., Chao L., Lei Z., Fashui H. (2009). Effects of Mg^2+^ on spectral characteristics and photosynthetic functions of spinach photosystem II. Spectrochim. Acta Part A Mol. Biomol. Spectrosc..

[37] Sun O.J., Gielen G.J.H.P., Sands R., Smith C.T., Thorn A.J. (2001). Growth, Mg nutrition and photosynthetic activity in *Pinus radiata*: Evidence that NaCl addition counteracts the impact of low Mg supply. Trees.

[38] Hariadi Y., Shabala S. (2004). Screening broad beans (*Vicia faba*) for magnesium deficiency. II. Photosynthetic performance and leaf bioelectrical responses. Funct. Plant Biol..

[39] Lasa B., Frechilla S., Aleu M., González-Moro B., Lamsfus C., Aparicio-Tejo P.M. (2000). Effects of low and high levels of magnesium on the response of sunflower plants grown with ammonium and nitrate. Plant Soil.

[40] Balakrishnan K., Rajendran C., Kulandaivelu G. (2000). Differential responses of iron, magnesium, and zinc deficiency on pigment composition, nutrient content, and photosynthetic activity in tropical fruit crops. Photosynthetica.

[41] Ceppi M.G., Oukarroum A., Çiçek N., Strasser R.J., Schansker G. (2012). The IP amplitude of the fluorescence rise OJIP is sensitive to changes in the photosystem I content of leaves: A study on plants exposed to magnesium and sulfate deficiencies, drought stress and salt stress. Physiol. Plant..

[42] Lavon R., Salomon R., Goldschmidt E.E. (1999). Effect of potassium, magnesium, and calcium deficiencies on nitrogen constituents and chloroplast components in Citrus leaves. J. Am. Soc. Hortic. Sci..

[43] Hörtensteiner S. (2009). Stay-green regulates chlorophyll and chlorophyll-binding protein degradation during senescence. Trends Plant Sci..

[44] Ren G., An K., Liao Y., Zhou X., Cao Y., Zhao H., Ge X., Kuai B. (2007). Identification of a novel chloroplast protein *AtNYE1* regulating chlorophyll degradation during leaf senescence in Arabidopsis. Plant Physiol..

[45] Wei Q., Guo Y., Kuai B. (2011). Isolation and characterization of a chlorophyll degradation regulatory gene from tall fescue. Plant Cell Rep..

[46] Tommasini R., Vogt E., Fromenteau M., Hörtensteiner S., Matile P., Amrhein N., Martinoia E. (1998). An ABC-transporter of *Arabidopsis thaliana* has both glutathione-conjugate and chlorophyll catabolite transport activity. Plant J..

[47] Boxler-Baldoma C., Lütz C., Heumann H.-G., Siefermann-Harms D. (2006). Structural changes in the vascular bundles of light-exposed and shaded spruce needles suffering from Mg deficiency and ozone pollution. J. Plant Physiol..

[48] Hermans C., Hammond J.P., White P.J., Verbruggen V. (2006). How do deficiencies of essential mineral elements alter biomass allocation. Trends Plant Sci..

[49] Igamberdiev A.U., Kleczkowski L.A. (2001). Implications of adenylate kinase-governed equilibrium of adenylates on contents of free magnesium in plant cells and compartments. Biochem. J..

[50] Puech L., Mehne-Jakobs B. (1997). Histology of magnesium-deficient Norway spruce needles influenced by nitrogen source. Tree Physiol..

[51] Wingler A., Brownhill E., Pourtau N. (2005). Mechanisms of the light-dependent induction of cell death in tobacco plants with delayed senescence. J. Exp. Bot..

[52] Scandalios J. (2005). Oxidative stress: Molecular perception and transduction of signals triggering antioxidant gene defenses. Braz. J. Med Biol. Res..

[53] Tewari R.K., Kumar P., Sharma P.N. (2006). Magnesium deficiency induced oxidative stress and antioxidant responses in mulberry plants. Sci. Hortic..

[54] Cai Y.-T., Zhang H., Qi Y.P., Ye X., Huang Z.-R., Guo J.-X., Chen L.-S., Yang L.-T. (2019). Responses of reactive oxygen species and methylglyoxal metabolisms to magnesium-deficiency differ greatly among the roots, upper and lower leaves of *Citrus sinensis*. BMC Plant Biol..

[55] Da Silva D.M., Brandão I.R., Alves J.D., Santos M.O., Souza K.R.D., Silveiri H.R.O. (2014). Physiological and biochemical impacts of magnesium-deficiency in two cultivars of coffee. Plant Soil.

[56] Candan N., Tarhan L. (2003). Relationship among chlorophyll-carotenoid content, antioxidant enzyme activities and lipid peroxidation levels by Mg^2+^ deficiency in the *Mentha pulegium* leaves. Plant Physiol. Biochem..

[57] Tewari R.K., Kumar P., Tewari N., Srivastava S., Sharma P.N. (2004). Macronutrient deficiencies and differential antioxidant responses-influence on the activity and expression of superoxide dismutase in maize. Plant Sci..

[58] Yu-Chuan D., Chang C.R., Luo W., Wu Y.-S., Ren X.-L., Wang P., Xu G.-H. (2008). High potassium aggravates the oxidative stress induced by magnesium deficiency in rice leaves. Pedosphere.

[59] Cakmak I., Marschner H. (1992). Magnesium deficiency and high light intensity enhance activities of superoxide dismutase, ascorbate peroxidase, and glutathione reductase in bean leaves. Plant Physiol..

[60] Ishijima S., Uchibori A., Takagi H., Maki R., Ohnishi M. (2003). Light-induced increase in free Mg^2+^ concentration in spinach chloroplasts: Measurement of free Mg^2+^ by using a fluorescent probe and necessity of stromal alkalinization. Arch. Biochem. Biophys..

[61] Gout E., Rébeillé F., Douce R., Bligny R. (2014). Interplay of Mg^2+^, ADP, and ATP in the cytosol and mitochondria: Unravelling the role of Mg^2+^ in cell respiration. Proc. Natl. Acad. Sci. USA.

[62] Moomaw A.S., Maguire M.E. (2008). The unique nature of Mg^2+^ channels. Physiology.

[63] Gardner R.C. (2003). Genes for magnesium transport. Curr. Opin. Plant Biol..

[64] Graschopf A., Stadler J.A., Hoellerer M.K., Eder S., Sieghardt M., Kohlwein S.D., Schweyen R.S. (2001). The yeast plasma membrane protein Alr1 controls Mg^2+^ homeostasis and is subject to Mg^2+^-dependent control of its synthesis and degradation. J. Biol. Chem..

[65] Li L., Tutone A.F., Drummond R.S.M., Gardner R.C., Luan S. (2001). A novel family of magnesium transport genes in Arabidopsis. Plant Cell.

[66] Gregan J., Kolisek M., Schweyen R.J. (2001). Mitochondrial Mg^2+^ homeostasis is critical for group II intron splicing in vivo. Genes Dev..

[67] Sponder G., Svidová S., Khan M.B., Kolisek M., Schweyen R.J., Carugo O., Djinović-Carugo K. (2013). The GMN motif determines ion selectivity in the yeast magnesium channel *Mrs2p*. Metallomics.

[68] Pisat N.P., Pandey A., MacDiarmid C.W. (2009). *MNR2* regulates intracellular magnesium storage in *Saccharomyces cerevisiae*. Genetics.

[69] Li H., Du H., Huang K., Chen X., Liu T., Gao S., Liu H., Tang Q., Rong T., Zhang S. (2016). Identification, and functional and expression analyses of the *CorA/MRS2/MGT*-type magnesium transporter family in maize. Plant Cell Physiol..

[70] Long A., Zhnag J., Yang L.-T., Ye X., Lai N.-W., Tan L.-L., Lin D., Chen L.-S. (2017). Effects of low pH on photosynthesis, related physiological parameters, and nutrient profiles of citrus. Front. Plant Sci..

[71] Schock I., Gregan J., Steinhauser S., Schweyen R., Brennicke A., Knoop V. (2000). A member of a novel *Arabidopsis thaliana* gene family of candidate Mg^2+^ ion transporters complements a yeast mitochondrial group II intron-splicing mutant. Plant J..

[72] Gebert M., Meschenmoser K., Svidova S., Weghuber J., Schweyen R., Eifler K., Lenz H., Weyand K., Knoop V. (2009). A root-expressed magnesium transporter of the *MRS2/MGT* gene family in *Arabidopsis thaliana* allows for growth in low-Mg^2+^ environments. Plant Cell.

[73] Chen Z.C., Peng W.T., Li J., Liao H. (2018). Functional dissection and transport mechanism of magnesium in plants. Semin. Cell Dev. Biology.

[74] Waters B.M. (2011). Moving magnesium in plant cells. New Phytol..

[75] Bose J., Babourina O., Rengel Z. (2011). Role of magnesium in alleviation of aluminium toxicity in plants. J. Exp. Bot..

[76] Chen Z.C., Yamaji N., Horie T., Che J., Li J., Gynheung A., Ma J.F. (2017). A magnesium transporter *OsMGT1* plays a critical role in salt tolerance in rice. Plant Physiol..

[77] Xu X.F., Wang B., Lou Y., Han W.-H., Lu J.-Y., Li D.-D., Li L.-G., Zhu J., Yang Z.N. (2015). Magnesium transporter 5 plays an important role in Mg transport for male gametophyte development in Arabidopsis. Plant J..

[78] Jones M.G., Outlaw W.H., Lowry O.H. (1977). Enzymic assay of 10^−7^ to 10^−14^ moles of sucrose in plant tissues. Plant Physiol..

[79] Smith R.L., Szegedy M.A., Kucharski L.M., Walker C., Weit R.M., Redpath A., Kaczmarek M.T., Maguire M.E. (1998). The CorA Mg^2+^ Transport Protein of *Salmonella typhimurium* Mutagenesis of conserved residues in the third membrane domain identifies a Mg^2+^ pore. J. Biol. Chem..

[80] Townsend D.E., Esenwine A.J., George J., Bross D., Maguire M.E., Smith R.L. (1995). Cloning of the *MgtE* Mg^2+^ transporter from *Providencia stuartii* and the distribution of *MgtE* in gram-negative and gram-positive bacteria. J. Bacteriol..

[81] Maguire M.E. (1992). MgtA and MgtB: Prokaryotic P-type ATPases that mediate Mg^2+^ influx. J. Bioenerg. Biomembr..

[82] Tao T., Snavely M.D., Farr S.G., Maguire M.E. (1995). Magnesium transport in *Salmonella typhimurium*: MgtA encodes a P-type ATPase and is regulated by Mg^2+^ in a manner similar to that of the MgtB P-type ATPase. J. Bacteriol..

[83] Snavely M., Florer J.B., Miller C.G., Maguire M.E. (1989). Magnesium transport in *Salmonella typhimurium*: ^28^Mg^2+^ transport by the CorA, MgtA, and MgtB systems. J. Bacteriol..

[84] Snavely M., Miller C., Maguire M. (1991). The *MgtB* Mg^2+^ transport locus of *Salmonella typhimurium* encodes a P-type ATPase. J. Biol. Chem..

[85] Smith R.L., Thompson L.J., Maguire M.E. (1995). Cloning and characterization of *MgtE*, a putative new class of Mg^2+^ transporter from *Bacillus firmus OF4*. J. Bacteriol..

[86] Chamnongpol S., Groisman E.A. (2002). Mg^2+^ homeostasis and avoidance of metal toxicity. Mol. Microbiol..

[87] Dann C.E., Wakeman C.A., Sieling C.L., Baker S.C., Irnov I., Winkler W.C. (2007). Structure and mechanism of a metal-sensing regulatory RNA. Cell.

[88] Li L.-G., Sokolov L.N., Yang Y.-H., Li D.-P., Ting J., Pandy G.K., Luan S. (2008). A mitochondrial magnesium transporter functions in Arabidopsis pollen development. Mol. Plant.

[89] Chen J., Li L.G., Liu Z.H., Yuan Y.J., Guo L.L., Mao D.D., Tian L.F., Chen L.B., Luan S., Li D.P. (2009). Magnesium transporter *AtMGT9* is essential for pollen development in Arabidopsis. Cell Res..

[90] Kobayashi N., Iwata N., Saito T. (2013). Application of ^28^Mg for characterization of Mg uptake in rice seedling under different pH conditions. J. Radio Anal. Nucl. Chem..

[91] Tanoi K., Kobayashi N.I., Saito T., Iwata N., Kamada R., Iwata R., Suzuki H., Hirose A., Ohmae Y., Sugita R. (2014). Effects of magnesium deficiency on magnesium uptake activity of rice root, evaluated using ^28^Mg as a tracer. Plant Soil.

[92] Mao D., Chen J., Tian L., Liu Z., Yang L., Tang R., Li J., Lu C., Yang Y., Shi J. (2014). Arabidopsis transporter *MGT6* mediates magnesium uptake and is required for growth under magnesium limitation. Plant Cell.

[93] Shaul O., Hilgemann D.W., Janice A.E., Montagu M.V., Inzé D., Galili D. (1999). Cloning and characterization of a novel Mg^2+^/H^+^ exchanger. Embo J..

[94] Yamaji N., Ma J.F. (2014). The node, a hub for mineral nutrient distribution in graminaceous plants. Trends Plant Sci..

[95] Drummond R., Tutone A., Li Y.C., Gardner R.C. (2006). A putative magnesium transporter *AtMRS2–11* is localized to the plant chloroplast envelope membrane system. Plant Sci..

[96] Marschner P., Marschner H. (1995). Mineral Nutrition of Higher Plants.

[97] Shaul O. (2002). Magnesium transport and function in plants: The tip of the iceberg. Biometals.

[98] Han S., Chen L.-S., Jiang H.-X., Smith B.-R., Yang L.-T., Xie C.-Y. (2008). Boron deficiency decreases growth and photosynthesis, and increases starch and hexoses in leaves of citrus seedlings. J. Plant Physiol..

[99] Hoagland D.R., Arnon D.I. (1950). The water-culture method for growing plants without soil. Circ. Calif. Agric. Exp. Stn..

[100] Lenz H., Dombinov V., Dreistein J., Reinhard R.M., Gebert M., Knoop V. (2013). Magnesium deficiency phenotypes upon multiple knockout of *Arabidopsis thaliana MRS2* clade B genes can be ameliorated by concomitantly reduced calcium supply. Plant Cell Physiol..

[101] Kobayashi N.I., Saito T., Iwata N., Ohmae Y., Iwata R., Tanoi K., Nakanishi T.M. (2013). Leaf senescence in rice due to magnesium deficiency mediated defect in transpiration rate before sugar accumulation and chlorosis. Physiol. Plant..

[102] Guo W. (2017). Magnesium homeostasis mechanisms and magnesium use efficiency in plants. Plant Macronutrient Use Efficiency, Molecular and Genomic Perspectives in Crop Plants.

[103] David-Assael O., Irina B., Shoshani-Knaani N., Helen S., Mizrachy-Dagri T., Jianxin C., Emil B., Shaul O. (2006). *AtMHX* is an auxin and ABA-regulated transporter whose expression pattern suggests a role in metal homeostasis in tissues with photosynthetic potential. Funct. Plant Biol..

[104] David-Assael O., Saul H., Saul V., Mizrachy-Dagri T., Berezin I., Brook E., Shaul O. (2005). Expression of *AtMHX*, an Arabidopsis vacuolar metal transporter, is repressed by the 5′ untranslated region of its gene. J. Exp. Bot..

[105] Akua T., Berezin I., Shaul O. (2010). The leader intron of *AtMHX* can elicit, in the absence of splicing, low-level intron-mediated enhancement that depends on the internal intron sequence. BMC Plant Biol..

[106] Haydon M.J., Román Á., Arshad W. (2015). Nutrient homeostasis within the plant circadian network. Front. Plant Sci..

[107] Werner T., Nehnevajova E., Kollmer I., Novak O., Strnad M., Kramer U., Schmulling T. (2010). Root-specific reduction of cytokinin causes enhanced root growth, drought tolerance, and leaf mineral enrichment in Arabidopsis and tobacco. Plant Cell.

[108] Niu Y., Chai R., Liu L., Jin G., Liu M., Tang C., Zhang Y. (2014). Magnesium availability regulates the development of root hairs in *Arabidopsis thaliana* (L.) Heynh. Plant Cell Environ..

[109] Malvi U.R. (2011). Interaction of micronutrients with major nutrients with special reference to potassium. Karnataka J. Agric. Sci..

[110] Bergmann W. (1992). Colour Atlas: Nutritional Disorders of Plants Development, Visual and Analytical Diagnosis.

[111] Farhat N., Rabhi M., Falleh H. (2013). Interactive effects of excessive potassium and Mg deficiency on safflower. Acta Physiol. Plant..

[112] Britto D.T., Kronzucker H.J. (2008). Cellular mechanisms of potassium transport in plants. Physiol. Plant..

[113] Schimansky C. (1981). The influence of certain experimental parameters on the flux characteristics of Mg-28 in the case of barley seedlings in hydroculture experiments. Landwirtsch. Forsch..

[114] Kleiber T., Golcz A., Krzesiński W. (2012). Effect of magnesium nutrition of onion (*Allium cepa* L.). Part I. Yielding and nutrient status. Ecol. Chem. Eng. S.

[115] Li Y., Han M.-Q., Lin F., Ten Y., Lin J., Zhu D.-H., Guo P., Weng Y.-B., Chen L.-S. (2015). Soil chemical properties, ‘Guanximiyou’ pummelo leaf mineral nutrient status and fruit quality in the southern region of Fujian province, China. J. Soil Sci. Plant Nutr..

[116] Moss G., Higgins M. (1974). Magnesium influences on the fruit quality of sweet orange (*Citrus sinensis* L. Osbeck). Plant Soil.

[117] Ye X., Chen X.-F., Deng C.-L., Yang L.-T., Lai N.-W., Guo J.-X., Chen L.-S. (2019). Magnesium-Deficiency Effects on Pigments, Photosynthesis and Photosynthetic Electron Transport of Leaves, and Nutrients of Leaf Blades and Veins in *Citrus sinensis* Seedlings. Plants.

[118] Löhnis M.P. (1960). Effect of magnesium and calcium supply on the uptake of manganese by various crop plants. Plant Soil.

[119] Bedi A., Sekhon G. (1977). Effect of potassium and magnesium application to soils on the dry-matter yield and cation composition of maize. J. Agric. Sci..

[120] Mikkelsen R. (2010). Soil and fertilizer magnesium. Better Crop..

[121] Van der Pol F., Traore B. (1993). Soil nutrient depletion by agricultural production in Southern Mali. Fertil. Res..

[122] Wang Z., Mahmood H.U., Faisal N., Liangquan W., Fusuo Z., Xuexian L. (2020). Magnesium Fertilization Improves Crop Yield in Most Production Systems: A Meta-Analysis. Front. Plant Sci..

[123] Mohebbi S., Mahler R. (1989). The effect of soil pH on wheat and lentils grown on an agriculturally acidified northern Idaho soil under greenhouse conditions. Commun. Soil Sci. Plant Anal..

[124] Guo J., Vogt R.D., Zhang X., Zhang Y., Seip H.M., Tang H. (2004). Ca-H-Al exchanges and aluminium mobility in two Chinese acidic forest soils: A batch experiment. Environ. Geol..

[125] Zhu M., Jiang X., Ji G. (2004). Experimental investigation on aluminum release from haplic acrisols in southeastern China. Appl. Geochem..

[126] Nguyen B.T., Thanh K.D., Thanh V.T., Mui K.D., Curtis J.D., Phuc V.L., Quyen T.K. (2018). High soil Mn and Al, as well as low leaf P concentration, may explain for low natural rubber productivity on a tropical acid soil in Vietnam. J. Plant Nutr..

[127] Hermans C., Simon J.C., Jiugeng C., Qiying X., Verbruggena N. (2013). An update on magnesium homeostasis mechanisms in plants. Metallomics.

[128] Lafond J. (2014). Fertilisation calcique et magnésienne dans la production du bleuet nain sauvage au Québec. Can. J. Soil Sci..

[129] Lixian Y., Zhou X., Peng Z., Chen W. (2005). Nutritional characteristics and K and Mg fertilizer combination in Baxi banana. Plant Nutr. Fertitizer Sci..

[130] Chen H.-b., Fan X.-l. (2018). Effects of magnesium remobilization and allocation on banana plant growth. J. Plant Nutr..

[131] Nasreen S., Ahmed R., Ullah M.A., Hoque M.A. (2013). Effect of N, P, K, and Mg application on yield and fruit quality of Mandarin (*Citrus reticulata*). Bangladesh J. Agric. Res..

[132] Li G.-l., Yao L.-X., Zhou X.-C., Zhang Y.-C., Tu S.-H. (2007). Effect of combination of K and Mg on Shatian pumelo. Soil Fertil. Sci. China.

[133] Lin X.-J., Li Y., Li Q., Wang F., Chunmei H. (2005). Effects of applying sulphate-potassium magnesium on yield and quality of pakchoi, tea and watermelon. Soils Fertil..

[134] Cao J. (2008). Calcium and Magnesium Nutrients Diagnosis Index in Soil and Effect of Fertilizing Calcium and Magnesium on the Growth of Chinese Cabbage. Chin. J. Soil Sci..

[135] Harris K., Vanajah T., Puvanitha S. (2018). Effect of foliar application of Boron and Magnesium on growth and yield of green chilli (*Capsicum annum* L.). Agrieast J. Agric. Sci..

[136] Poberezny J., Wszelaczynska E., Keutgen A.J. (2012). Yield and chemical content of carrot storage roots depending on foliar fertilization with magnesium and duration of storage. J. Elem..

[137] Bolton J., Penny A. (1968). The effects of potassium and magnesium fertilizers on yield and composition of successive crops of ryegrass, clover, sugar beet, potatoes, kale and barley on sandy soil at Woburn. J. Agric. Sci..

[138] Laxminarayana K., Susan J.K., Ravindran C.S., Naskar S.K. (2011). Effect of lime, inorganic, and organic sources on soil fertility, yield, quality, and nutrient uptake of sweet potato in alfisols. Commun. Soil Sci. Plant Anal..

[139] Zengin M., Gökmen F., Gezgin S., Cakmak I. (2008). Effects of different fertilizers with potassium and magnesium on the yield and quality of potato. Asian J. Chem..

[140] Huang J.-C., Peng Z., Yu J., Lin Z., Wu X., Yang L. (2014). The effect of different magnesium fertilizer rates on the yield and quality of winter potatoes. Guangdong Agric. Sci..

[141] Orlovius K., McHoul J. (2015). Effect of two magnesium fertilizers on leaf magnesium concentration, yield, and quality of potato and sugar beet. J. Plant Nutr..

[142] Fajemilehin S., Babayemi O., Fagbuaro S. (2008). Effect of anhydrous magnesium sulphate fertilizer and cutting frequency on yield and chemical composition of *Panicum maximum*. Afr. J. Biotechnol..

[143] Ruan J., Guan Y., Wu X. (2002). Status of Mg availability and the effects of Mg application in tea fields of Red Soil area in China. Sci. Agric. Sin..

[144] Ceylan Y., Kutman U.B., Mengutay M., Cakmak I. (2016). Magnesium applications to growth medium and foliage affect the starch distribution, increase the grain size and improve the seed germination in wheat. Plant Soil.

[145] Ertiftik H., Zengin M. (2017). Response of maize for grain to potassium and magnesium fertilizers in soils with high lime contents. J. Plant Nutr..

[146] Zeng Z., Liao Q., Wu Y., Li X. (2012). Effects of magnesium fertilizer on agronomic characters and yield of rice. Mod. Agric. Sci. Technol..

[147] He T., Wei J., Huang H. (1997). Effect of magnesium fertilizer on yield and quality of sugarcane. J. South Agric..

[148] Ertiftik H., Zengin M. (2016). Response of sunflower to potassium and magnesium fertilizers in calcerous soils in central Anatolia of Turkey. J. Plant Nutr..

[149] Cwalina-Ambroziak B., Wierzbowska J., Damszel M., Bowszys T. (2012). The effect of mineral fertilization on achenes yield and fungal communities isolated from the stems of milk thistle *Silybum marianum* (L.) Gaertner. Acta Sci. Pol. Hortorum. Cultus.

[150] Azizi K., Yagobhi M., Hidary S., Chaechi R.M., Roham R. (2011). Effects of different methods of magnesium sulphate application on qualitative and quantitative yield of lentil (*Lens culinaris* Medik.) cultivars under Khorramabad climatic conditions of Iran. Res. Crop..

[151] Huazhang X., Zhou Y., Fan Q., Zhong Y. (2003). The Effect of Spray Magnesium Fertilizer to Peanut Planted in Krasnozem. Chin. Agric. Sci. Bull..

[152] Yan S., Pentao L. (1992). Effect of Magnesium to Output. qualities and some physiological Indices of Flued-tobacco. J. Yunnan Agric. Univ..

[153] Xu Q., Chen A., Dai P., Zheng G., Chen Z. (2011). Effects of Rational Application of Magnesium Fertilizers on Growth, Yield and Quality of Flue-cured Tobacco. Chin. Tob. Sci..

[154] Li Y.-Z., Jiang Z.-H., Yang Z.-X., Luo P.-T. (2002). Effects of Magnesium Supply on main economic characters in Flue-Cured Tobacco. J. Southwest Agric. Univ..

[155] Deng W., Luo K., Li D., Zheng X., Wei X., Smith W., Thammina C., Lu L., Li Y., Pei Y. (2006). Overexpression of an Arabidopsis magnesium transport gene, *AtMGT1*, in *Nicotiana benthamiana* confers Al tolerance. J. Exp. Bot..

[156] White P.J., Broadley M.R. (2009). Biofortification of crops with seven mineral elements often lacking in human diets–iron, zinc, copper, calcium, magnesium, selenium and iodine. New Phytol..

[157] Jezek M., Geilfus C.M., Bayer A., Mühling K.H. (2015). Photosynthetic capacity, nutrient status, and growth of maize (*Zea mays* L.) upon MgSO_4_ leaf-application. Front. Plant Sci..

[158] Broadley M.R., Hammond J.P., King G.J., Bowen H.C., Hayden R., Spracklen W.P., Ó Lochlainn S., White P.J. (2009). Biofortifying Brassica with calcium (Ca) and magnesium (Mg). https://escholarship.org/uc/item/9936g2vv.

[159] Kashinath B.L., Murthy A.G., Senthivel T., Pitchai G.J., Sadashiva A.T. (2013). Effect of applied magnesium on yield and quality of tomato in Alfisols of Karnataka. J. Hortic. Sci..

[160] Babaeian M., Esmaeilian Y., Tavassoli A., Asgharzade A. (2012). Efficacy of different iron, zinc and magnesium fertilizers on yield and yield components of barley. Afr. J. Microbiol. Res..

[161] Özenç N., Özenç D.B. (2015). Effect of magnesium fertilization on some plant nutrient interactions and nut quality properties in Turkish hazelnut (*Corylus avellana* L.). Sci. Res. Essays.

[162] Broadley M.R., Hammond J.P., King G.J., Astley D., Bowen H.C., Meacham M.C., Mead A., Pink D.A.C., Teakle G.R., Hayden R.M. (2008). Shoot calcium and magnesium concentrations differ between subtaxa, are highly heritable, and associate with potentially pleiotropic loci in *Brassica oleracea*. Plant Physiol..

[163] Alexander E.B., Robert G.C., Harrison S.P. (2007). Serpentine Geoecology of Western North America: Geology, Soils, and Vegetation.

[164] Turner T.L., Elizabeth C., Eric J., Tina T.H., Sergey V.N. (2010). Population resequencing reveals local adaptation of *Arabidopsis lyrata* to serpentine soils. Nat. Genet..

